# Supplementary material for: “When the Word Is Too Big, It’s Just Too Hard”: Stroke Survivors’ Perspectives About Health Literacy and Delivery of Health Information
Source: Healthcare (Basel). 2025 Mar 3;13(5):541. doi: 10.3390/healthcare13050541 (PMC11898806; doi:10.3390/healthcare13050541)
Supplement: Supplementary file 1 [file healthcare-13-00541-s001.zip › healthcare-3473131-supplementary.pdf]

Vignettes

Cluster 1

Anne is a 51 year old female who lives with her family; a husband and two children in their early twenties. Her family are supportive and she has many good friends.

Before her stroke, Anne worked part time as a bookkeeper. She liked her work but has not been able to return to it after her stroke. She has used up all of her sick leave and will soon start to run out of her long service leave. She is quite anxious about what will happen about this in the long term.

Anne has high cholesterol that she did not know about before her stroke. She now takes medication to help manage this. Otherwise she has no other medical history before her stroke, her GP was the only health professional she saw for her annual check-up. She has always been fit and active, before her stroke she would walk three times a week with a friend and go to a weekly yoga class. She has started going back for walks with her friend again but has not yet returned to her yoga class.

Anne has had very limited experience of the health system prior to her stroke. Because of this lack of experience she doesn't know where to go for help. She is finding it hard to know what services are out there to help her get back to some kind of work, and also what questions she should ask the specialist. She is worried the specialists will think her questions are silly and although she likes her GP she thinks they are very busy and doesn't want to make unnecessary appointments to discuss her concerns with them.

Cluster 2

Antonio is a 63 year old male who was born in Italy. He moved to Australia with his parents when he was six years old. He lives alone and has a good relationship with his ex-wife and two adult children who live close by.

Before his stroke Antonio worked as metal fabricator. He wants to return to work but the ongoing problems from his stroke mean that this has not been possible. He misses the social aspect of his work, but even before his stroke he was finding the physical demands of his job hard.

Antonio smokes 20 cigarettes a day and has diabetes and high cholesterol. He knows he needs to make some changes to his lifestyle to prevent another stroke; the problem is, is doesn't know where to start. He tried to talk to his diabetes specialist at his last appointment but found the information hard to understand. He felt he had forgotten a lot of it once the appointment ended. Antonio may have functional spoken English but has difficulty understanding information which is more complex than social conversation. He should be offered an interpreter to assist with medical consultations even if he declines.

His children are supportive and take it in turns to bring him to the hospital for appointments. After the appointment they talk to him about what was discussed with the specialist and help him make decisions about his medical treatment.

Antonio received a 'My Stroke Journey' pack when he was first diagnosed with a stroke in hospital. He found some of the information helpful, but there was so much of it that it started to overwhelm him. He asked one of the doctors about it in hospital but they used lots of medical words which left him feeling more confused. Antonio might have limited literacy in both English and Italian.

Cluster 3

Mai is a 75 year old female who moved to Australia in the 1970s. She was born in Vietnam and only speaks Vietnamese. She was unable to finish her secondary school education and worked in a factory for most of her life. Mai's husband died two years ago, her main support is now her daughter who lives close by but works long hours and has two small children.

Because of the COVID-19 pandemic Mai was unable to have visitors while she was in hospital after her stroke. She found this very scary and a lot of the time was unsure what was happening. Her daughter spoke to the doctors each day about her, but she's not sure what they spoke about. None of the doctors or nurses could speak Vietnamese, nor was she offered an interpreter so she was unable to ask any questions. The Vietnamese language leaflets she was given didn't always make sense to her, she thinks that they probably wouldn't have helped her anyway.

Mai has a good GP, he speaks Vietnamese and Vietnamese and has some familiarity with the language. This helps Mai in trusting him in discussing her problems. Because her daughter has young children and a busy job, she can't always come to Mai's specialist appointments with her. She likes appointments where she has an interpreter, this means she can ask questions. She prefers to hear verbal information with an interpreter rather than have it in writing as she has limited literacy in Vietnamese. It also gives her chance to socialise, since having her stroke she doesn't get to see people from her community very much anymore.

Mai takes lots of medications and had lots of tests after her stroke. She's not sure what the medications and tests are for, but the doctor told her they are important. Her doctors have told her to exercise more and change her diet, Mai likes the food she cooks and walks round the block every other day. She doesn't think she needs to change this as she is taking the tablets the doctor told her to.

Cluster 4

Michael is a 59 year old male and lives alone having recently separated from his partner. He works long hours running his own business which means he doesn't have much time to socialise or take care of his health.

After his stroke Michael was able to return to working for himself and still works very long hours. He knows he needs do to more look after himself after his stroke, he thinks he was lucky that he has made a good recovery.

Michael tried to do research about his stroke on the internet but there was a lot of conflicting information. This made it hard for him to make plans about what he should to reduce his stroke risk. He's still not exactly sure why he had the stroke but thinks his lifestyle might have something to do with it.

At his last stroke clinic appointment he felt like he a good chat with his stroke specialist. He was able to ask lots of questions and asked the doctor to refer him to a dietician to help with weight loss. His first appointment with the dietician was via telehealth. They emailed him lots of information about how to read food labels but Michael finds talking to people and practising things in real life a much easier way to learn.
